# EcoRxChoice.com— an SIDP-funded calculator integrating environmental metrics with antimicrobial stewardship

**DOI:** 10.1017/ash.2026.10398

**Published:** 2026-07-06

**Authors:** Pamela S. Lee, Hugh Gordon, Theresa Ferguson, Tien Dinh, Misty Vu, Marina Nguyen, Chaynor Hsiao, Gary Fong

**Affiliations:** 1 Department of Medicine, David Geffen School of Medicine at ULCA, Los Angeles, CA, USA; 2 Department of Medicine, Division of Infectious Diseases, Harbor-UCLA-Medical Center, Torrance, CA, USA; 3 Department of Medicine, Los Angeles General Medical Center, USA; 4 Department of Medicine, Keck School of Medicine at the University of Southern California, USA; 5 Society of Infectious Diseases Pharmacists, USA; 6 School of Pharmacy, Chapman University, Orange, CA, USA; 7 EcoRxChoice.com, USA

## Abstract

Healthcare sustainability is a multidisciplinary field that seeks to mitigate healthcare’s environmental consequences. Antimicrobial stewardship and healthcare sustainability both aim to reduce wasteful resource use while maximizing patient safety. However, a strong partnership between antimicrobial stewardship and healthcare sustainability has yet to develop. To facilitate environmentally sustainable decision-making in antimicrobial prescribing, we developed a web-based calculator (EcoRxChoice (www.ecorxchoice.com)) where users can enter different antimicrobial regimens and compare how much plastic waste each regimen creates. This work was supported in part by the Society of Infectious Diseases Pharmacists (SIDP), reflecting SIDP’s commitment to strengthening sustainability as a practical extension of antimicrobial stewardship and empowering infectious disease pharmacists to make measurable environmental impact through evidence-based care.

## Leveraging stewardship programs for planetary health

Antimicrobial stewardship programs were created to protect patients today while preserving antimicrobial effectiveness for the future, and they increasingly intersect with the parallel priority of planetary health. Healthcare systems are being called upon to reduce waste and greenhouse gas emissions while maintaining the reliability and safety of clinical care. Yet medications, including antimicrobials, are an underappreciated driver of environmental impact. Beyond the drugs themselves, pharmaceutical waste includes non-biodegradable materials such as plastic wrapping, packing materials, and the high volume of single-use materials required for pharmacy operations. Such sources of waste are often invisible to clinicians who do not observe medication preparation and distribution processes. Stewardship programs are ideally positioned to bridge the current gap between antibiotic overuse and healthcare waste, as stewardship already focuses on minimizing unnecessary therapy, improving efficiency, and reducing downstream harm—all of which also reduce healthcare’s environmental impact. In turn, strategies that emphasize environmental stewardship may also help mitigate supply chain vulnerabilities by reducing demand, conserving critical resources, and promoting more resilient procurement practices.

Environmental sustainability and antimicrobial stewardship share a common foundation in resource stewardship, accountability, and population-level disease prevention. Stewardship teams routinely assess tradeoffs between efficacy, safety, resistance risk, feasibility, and cost. Adding environmental parameters to this framework preserves stewardship’s core clinical goals and strengthens decision-making when several therapeutic options are otherwise appropriate. Translating sustainability priorities into actionable prescribing decisions, however, requires tools that clinicians can use without disrupting workflow or introducing uncertainty into patient care. In this sense, sustainability in stewardship depends on practical strategies that can guide choices at the point of prescribing and at the level of systemwide practice.

## The environmental footprint of healthcare plastics

Health impacts from plastic chemicals are responsible for hundreds of billions of dollars of healthcare costs annually.^1^ Microplastics and nanoplastics in human tissues have been linked prospectively to health outcomes—carotid endarterectomy plaques containing microplastics were associated with increased mortality compared to plaques without microplastics.^2^ Plastic production leads to significant greenhouse gas emission generation and is projected to cause 4.5% of all global greenhouse gas emissions by 2060.^3^ Despite these human and planetary health impacts, production and use of single-use plastics continue to escalate globally.^3^

The healthcare sector is a significant contributor to plastic waste. U.S. hospitals produce over 5.9 million tons of waste each year, including 1.7 million tons of plastic waste.^4^ Furthermore, the U.S. healthcare sector causes disproportionately large greenhouse gas emissions and pollution, a significant amount of which are from pharmaceuticals.^5^ Antimicrobials are among the most commonly administered medications and are resource-intensive throughout their lifespan.^6,7^ Unused antimicrobial disposal causes ecosystem disruptions and increases environmental antimicrobial resistance.^8,9^ For intravenous antimicrobials, accompanying medical supplies further exacerbate healthcare-derived pollution.^10^

Healthcare sustainability is a multidisciplinary field that seeks to mitigate healthcare’s environmental consequences. Antimicrobial stewardship and healthcare sustainability both aim to reduce wasteful resource use while maximizing patient safety. However, a formal partnership between antimicrobial stewardship and healthcare sustainability has yet to develop.

## The integration of stewardship and sustainability

To facilitate environmentally sustainable decision-making in antimicrobial prescribing, we developed a web-based calculator (EcoRxChoice (www.ecorxchoice.com)) where users can enter different antimicrobial regimens and compare how much plastic waste each regimen creates. This work was supported in part by the Society of Infectious Diseases Pharmacists (SIDP), reflecting SIDP’s commitment to strengthening sustainability as a practical extension of antimicrobial stewardship and empowering infectious disease pharmacists to make measurable environmental impact through evidence-based care. Infectious disease pharmacists are ideally positioned to integrate sustainability and stewardship due to their clinical knowledge, understanding of the medication-use processes, and role in pharmaceutical purchasing.

Below, we describe the methods used to create this calculator as well as suggestions for use at both individual and organizational levels. EcoRxChoice offers a pragmatic framework for everyday clinicians that incorporates environmental metrics into antimicrobial stewardship decision-making without displacing established clinical priorities.

## Approach to quantifying plastic waste from antimicrobial regimens

We compiled a list of all inpatient antimicrobials available at our institution. Using package inserts, local IV compounding recipe formulas, and online databases, we identified and quantified all plastic material required to compound and administer each antimicrobial. Weights of plastic packaging and supplies used in the compounding and dispensing process were collected directly in the hospital using a scale accurate to 0.01 g (Table 1). We tabulated the component quantities and weights from each compounding recipe to calculate plastic waste generated per individual dose. This information was shared with a third-party programmer, who created the coding, algorithm, and web design for EcoRxChoice.

**Table 1.** List of supplies and associated weights Table 1 long description.

Item	Plastic (g)
Syringe	10.0
Needle cap	2.0
Needle hub	2.0
5-Micron filter	7.0
50 mL IVPB	28.1
100 mL IVPB	56.2
150 mL IVPB	84.3
250 mL IVPB	140.5
300 mL IVPB	168.6
500 mL IVPB	281.0
1,000 mL IVPB	562.0
IV Tubing	53.0
Flush	10.0
Admin cup	1.0
Unit dose ziploc	1.0
Blister card	0.4

**Figure 1.**
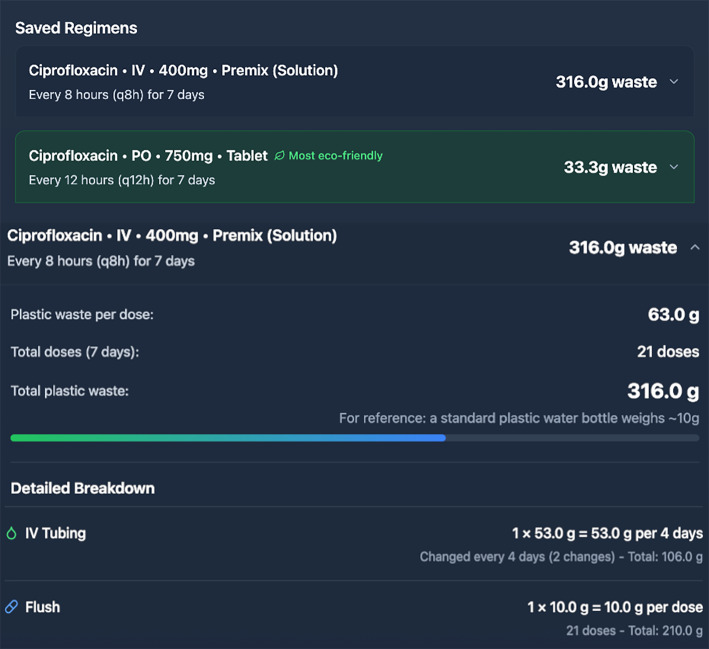
(a and b) Example output for EcoRxChoice.com.

Users of EcoRxChoice select an antimicrobial and associated dose, administration method, form, frequency, and duration of therapy. The calculator generates the plastic waste per dose as well as the plastic waste per regimen. Users may select “Save regimen” and compare their antimicrobial choices against other potential regimens. The regimen that generates the least plastic waste is identified as “most eco-friendly” (Figure 1a). Users may click on a “Detailed Breakdown” (Figure 1b) to determine what components of the antimicrobial regimen are logged as contributing to plastic waste to maximize transparency of the calculator’s output.

Notably, early versions of the EcoRxChoice calculator solely focused on plastic waste to simplify and focus its output. Key assumptions include that IV tubing is changed every 4 days—the maximum allowable time between changes at many institutions—to not overestimate plastic waste associated with IV antimicrobial regimens. In general, the calculator was designed to intentionally underestimate environmental impact.

## Use cases, early adoption, and future directions

EcoRxChoice was released for beta-testing in April 2025. Since we began tracking webpage analytics in December 2025, EcoRxChoice.com has logged on average 40 active users and 90 pageviews per week. Users have originated from 10 different countries, from which users from the US are most frequent. EcoRxChoice has been integrated into stewardship webpages and teaching curricula in multiple institutions. As adoption grows, EcoRxChoice will provide clinicians, educators, and stewardship teams with practical, data-informed insights at the point of care. Continued collaboration and feedback will help refine the platform and expand its impact, driving lasting progress in sustainable healthcare.

EcoRxChoice demonstrates how patient-level inputs can be translated into estimates of plastic waste at individual and system levels (Table 2). This same framework could be expanded to quantify “diagnostic waste” by assigning standardized measures to inputs from commonly overused diagnostic tests (eg, repeated blood, respiratory, and urine cultures). Embedding this into prospective workflows would allow stewardship teams to present real-time waste alongside traditional antibiotic recommendations. Patients/providers could be flagged not only for de-escalation of antibiotic therapy but also for redundant lab testing, with an estimation of downstream impact. At a systems level, waste calculators could be aggregated into dashboards showing trends in wasteful testing and opportunities for improvement, enabling regular reporting that links stewardship with financial, environmental, and patient safety metrics.

**Table 2.** Suggested use cases for EcoRxChoice Table 2 long description.

Use case	Description	Example(s)
Provide environmental parameters to inform antimicrobial selection	Assuming regimens are otherwise similar, clinicians may compare different antimicrobial choices in terms of plastic waste.	Cefazolin versus oxacillin for methicillin-susceptible *S. aureus* bloodstream infections
Reduce duration of antimicrobial therapy	Clinicians across specialties are enthusiastic about reducing the environmental impact of healthcare.^11^ EcoRxChoice can quantify plastic waste associated with longer durations of therapy and may increase non-ID clinician engagement in reducing length of therapy.	14-day versus 7-day antibiotic regimens for pneumonia
Promote IV to PO conversions of antibiotics	Oral antibiotics generate less plastic waste than IV, however IV antibiotics are frequently used even when highly bioavailable equivalents exist. Users can quantify plastic waste associated with unnecessary IV antibiotics and how much plastic waste could be mitigated by early IV to PO switches.	Oral versus IV fluoroquinolones; early PO switches for osteomyelitis or endocarditis
Inform dosing strategies	Antimicrobial stewardship programs considering dosing protocol changes may use EcoRxChoice to include environmental impact data in their cost-benefit analyses of such changes.	Twice-daily versus thrice-daily metronidazole

## Table 1 Long description

A table with two columns and fourteen rows. The first column is labeled Item and the second column is labeled Plastic (g). The table lists various medical items and their associated plastic weights in grams. Row 1: Syringe, 10.0. Row 2: Needle cap, 2.0. Row 3: Needle hub, 2.0. Row 4: 5-Micron filter, 7.0. Row 5: 50 mL IVPB, 28.1. Row 6: 100 mL IVPB, 56.2. Row 7: 150 mL IVPB, 84.3. Row 8: 250 mL IVPB, 140.5. Row 9: 300 mL IVPB, 168.6. Row 10: 500 mL IVPB, 281.0. Row 11: 1,000 mL IVPB, 562.0. Row 12: IV Tubing, 53.0. Row 13: Flush, 10.0. Row 14: Admin cup, 1.0. Row 15: Unit dose ziploc, 1.0. Row 16: Blister card, 0.4.

Navigate back to Table 1.

## Table 2 Long description

A table with three columns: Use case, Description, and Example(s). The table has four rows, each describing a different use case for EcoRxChoice. Row 1: Use case: Provide environmental parameters to inform antimicrobial selection. Description: Assuming regimens are otherwise similar, clinicians may compare different antimicrobial choices in terms of plastic waste. Example(s): Cefazolin versus oxacillin for methicillin-susceptible S. aureus bloodstream infections. Row 2: Use case: Reduce duration of antimicrobial therapy. Description: Clinicians across specialties are enthusiastic about reducing the environmental impact of healthcare. EcoRxChoice can quantify plastic waste associated with longer durations of therapy and may increase non-ID clinician engagement in reducing length of therapy. Example(s): 14-day versus 7-day antibiotic regimens for pneumonia. Row 3: Use case: Promote IV to PO conversions of antibiotics. Description: Oral antibiotics generate less plastic waste than IV, however IV antibiotics are frequently used even when highly bioavailable equivalents exist. Users can quantify plastic waste associated with unnecessary IV antibiotics and how much plastic waste could be mitigated by early IV to PO switches. Example(s): Oral versus IV fluoroquinolones; early PO switches for osteomyelitis or endocarditis. Row 4: Use case: Inform dosing strategies. Description: Antimicrobial stewardship programs considering dosing protocol changes may use EcoRxChoice to include environmental impact data in their cost-benefit analyses of such changes. Example(s): Twice-daily versus thrice-daily metronidazole.

Navigate back to Table 2.
